# Colour Transition Dynamics of Commercial Plant- and Animal-Based Meat Analogues

**DOI:** 10.3390/foods14213616

**Published:** 2025-10-23

**Authors:** Dhanushka Rathnayake, Jaqueline Moura Nadolny, Yasmina Sultanbawa, Heather Eunice Smyth

**Affiliations:** Centre for Nutrition and Food Sciences, Queensland Alliance for Agriculture and Food Innovation (QAAFI), The University of Queensland, Brisbane, QLD 4072, Australia; p.dhanushka@uq.edu.au (D.R.); j.mouranadolny@uq.edu.au (J.M.N.); y.sultanbawa@uq.edu.au (Y.S.)

**Keywords:** colour, plant-based meat analogues, Maillard reaction, browning

## Abstract

The inferior visual sensory attributes, particularly colour, of plant-based burgers, remain a barrier to enhancing consumer acceptance and uptake in the global market. This study aimed to comprehensively profile the colour transition dynamics at varying internal temperatures (uncooked, 35 °C, 55 °C, 75 °C, and 85 °C) of four distinct commercial plant-based (PB) and six animal-based (AB) burgers, and to identify key “*colour gaps*” for improvement. Raw beef burgers appeared red with higher positive a* values (redness), whereas v2food, vEEF, and Beyond burgers showed comparatively higher b* (yellowness) and c* (chroma) values both externally and internally. The sample Impossible PB burgers had the lowest colour differences (∆a*, ∆b*, ∆c*, and ∆E*), showing a beef-like colour transition in both raw and cooked states. Chicken and pork+beef burgers exhibited lower redness in the processed visual images attributed to higher L* values owing to lower myoglobin content. In AB burgers, a* was negatively correlated with L* and *h*°, while PB burgers positively correlated with b* and c*. The browning intensity observed in both AB and PB burgers is influenced by their internal structural characteristics, which respond dynamically to changes in internal temperature. Mapping the colour transition during the cooking of AB and PB burgers is a critical first step toward identifying gaps in PB product development. Enhancement of visual sensory attributes can be achieved through the modelling of suitable natural colour combinations to target specific dimensions in the colour space.

## 1. Introduction

As the global population is projected to reach approximately 10 billion by 2050, the demand for livestock production is predicted to double. This necessitates the development of nutritious, sustainable, and healthy meat substitutes to cater to the expanding population [1,2]. Livestock production, responsible for 18–56% of total greenhouse gas emissions, exerts a significant environmental challenge. A transition from animal-based to vegan diets could potentially reduce these emissions by 35–50% [3,4]. Moreover, excessive meat consumption has been linked to various health risks, including colorectal cancer, cardiovascular diseases, obesity, diabetes, and non-alcoholic fatty liver conditions [5]. In Australia, the average meat intake significantly surpasses global recommendations, raising health concerns [6]. Additionally, ethical issues arise from the substandard welfare conditions prevalent in the industrialized livestock sector [7]. The emergence of alternative protein sources, particularly plant-based (PB) meat, coupled with advancements in food technology, represents a rapidly expanding economic frontier. This sector is projected to achieve substantial market valuation by 2030, driving the development of innovative business models globally [8]. Plant-based meat analogues (PBMAs) have emerged as a sustainable solution to these pressing global issues, with the Australian plant-based meat sector projected to contribute approximately AUD 3 billion to the domestic market by 2030 [9].

Despite the numerous benefits of plant-based (PB) meat, imitating the flavour, nutritional profile, texture, and colour of animal-based (AB) meat presents challenges to the development of PBMAs for the industry [10]. In terms of sensory gaps, colour emerges as a key obstacle in PB meat analogue formulation; however, there are comparatively few studies addressing the colour dimensions that need to be enhanced, optimal pigment combinations, or effective incorporation methods within the matrix. [11,12]. Colour is a primary sensory property that affects consumers’ perception, initial acceptance, selection, or rejection of food products [9]. In meat, consumers rely heavily on external and internal colour, which conveys both the freshness of raw meat as well as the “*doneness*” of cooked meat [13,14]. Given the unique ingredient formulations of PBMAs and the absence of natural pigments like myoglobin in red meat, mimicking the visual appearance of red meat, both raw (reddish) and cooked (brownish), is a significant challenge. With the consumer push for more natural healthy ingredients, a new generation of commercial PBMAs has turned to natural colourants, such as betanin and anthocyanins in Beyond burgers, leghaemoglobin (LegHb) in Impossible burgers, betanin in v2food and vEEF burgers, lycopene and carrot extract in “Tofurky™”, and tomato paste, which contains high lycopene content, in “MorningStar Farms^TM^” [11]. The intense earthy flavour, undesirable yellow hue appearance, and colour stability of betanin and consumers’ reluctance to purchase products that have genetically modified colouring agents (e.g., LegHb) in commercial PB products have led to exploring novel colouring approaches [15]. The generation of heterocyclic amines identified as carcinogenic substances through the synthetic browning colouring agents in commercial products is another reason to investigate a healthier browning process in PB meat [16,17]. The identification of the colour-related discrepancies in current commercial PBMAs from meat-based burgers is of utmost importance when implementing a potentially promising pigment-based colour transition approach.

In the current study, varying endpoint internal temperatures were investigated in terms of how they influence the external and cross-sectional colour dispersion of commercially available PB and AB burgers. The main objectives involved setting up a processed imaging system and colour maps for both the external and internal view of burgers, with the aim of assessing the extent to which the current generation of PB burgers replicates the colour distribution of authentic animal-based burgers. This study will provide valuable insights into identifying the gaps that need to be addressed in the colour space to stimulate further research in developing high-quality meat analogues with authentic meat-like appearances using natural ingredients.

## 2. Materials and Methods

### 2.1. Sample Selection and Preparation

Ten commercially available AB (6 brands) and PB (4 brands) burgers were purchased as fresh chilled products from local supermarkets (Coles and Woolworths supermarkets in Brisbane, Australia) and controlled by the expiry date or batch number within a specific brand (Table 1). The samples were immediately stored (−19 °C) with their commercial packaging. When needed, the samples were thawed in a refrigerator (2 °C) for a duration of 24 h. An hour prior to cooking, the samples were removed to room temperature (22 °C). A Silex GTTPower save double grill (Silex Holdings Australia Pvt Ltd., Marrickville, NSW, Australia) was pre-heated, following which both PB and AB burgers were cooked at a plate temperature of 160 °C [18]. The cooking process continued until the internal temperature of the burger reached the set endpoint internal temperature of either 35 °C, 55 °C, 75 °C, or 85 °C, respectively. The internal temperature was measured using a digital thermometer inserted around 0.6 cm into the burger without contacting the hot grill surface (Single input thermometer w/recording—IC-FLUKE-53-2-B-50Hz, Fluke Corporation, Everett, WA, USA). All cooked samples were placed on unwrapped aluminium foil and allowed to cool for 5–10 min prior to photographing and further analysis.

At the time of this study, four different PB burgers imitating beef burgers were commercially available, and vegetable patties were not included in this study as they do not attempt to mimic meat. Similarly, certain beef-based burgers, which had been infused with various colourants, were excluded. The selection of AB burgers was based on representing a wide colour range due to myoglobin content in the muscle tissues: highest (kangaroo-due to oxidative muscle fibres), high (beef), low (pork and beef blend), and lowest (chicken) [19,20]. The commercial chicken burgers consist of spices and parsley as herbs to enhance appearance, flavour attributes, and subsequent consumers’ purchasing decisions. According to their nutritional composition (as displayed on the package), as provided in Table 1, the vEEF, and Classic beef burgers had the lowest protein content (~13 g/100 g), while other burgers ranged from 15 to 18 g per 100 g. Total fat content was similar for v2food and vEEF (13~15 g/100 g), with Beyond and Impossible showing the highest (19 g/100 g) and lowest (11 g/100 g) values among PB burgers, respectively. Notably, kangaroo burgers had the lowest fat content (1.9 g/100 g) among both PB and AB burgers, whereas Classic beef burgers had the highest fat content (21 g/100 g).

### 2.2. Digital Image Acquisition

Digital images of the cooled burgers at each temperature level were captured using a digital SLR camera (Canon 80D, Canon Inc., Tokyo, Japan) under uniform light conditions. The portable lightbox (Model THPHOSB1, LED photography studio box, THUNDA, Beijing, China) is composed of three vertical sides and a roof, totalling five sides, including a white LED light ring attached to the roof. A 9 cm diameter opening in the roof facilitates overhead photography of the samples, while a frontal opening allows for the arrangement and capture of the burgers’ cross-sectional view. The camera was positioned vertically above the background at a distance of 17.5 cm to capture an external view from above the burger. The cross-sectional photographs were taken from the front opening, 20 cm away from the burger. Prior to photography, exudates and oil on the surface of the burgers were meticulously wiped off using tissue paper to avoid inconsistent light scattering and potentially obscure true surface colour characteristics of burgers.

The interior walls of the lightbox were lined with a matte, white finishing foam board to prevent specular reflection. The burgers were placed on matte white tile (16 cm × 16 cm × 0.5 cm), accompanied by a measuring rule, and positioned in the centre of the lightbox. The lightbox’s lighting system consists of LED lamps (Natural daylight, 100 W), and photos were taken at a consistent height (camera settings: ISO 100; lens aperture at *f* = 3.7, no flash, maximum resolution 2048 × 1536 pixels) and stored in JPG format. The raw digital images underwent pre-processing, which included colour calibration and background removal, without compromising image quality, using Adobe Photoshop software premium version (2023).

### 2.3. Instrumental Colour Measurement

The effect of different cooking temperatures on colour variations (Commission Internationale de l’Éclairage [CIE Lab]) of the samples was analysed using a colourimeter in 10° viewing angle (Konica Minolta Chroma Meter CR-400, Osaka, Japan). The instrument was calibrated using a white plate (CR 400/C Y: 84.0, x: 0.3174, y: 0.3236). The value a* (redness) ranges from green (−) to red (+), L* (lightness) from black (−) to white (+), b* (yellowness) from blue (−) to yellow (+), c* the relative colour saturation (chroma), and *h*° the hue which units are in the form of degrees (or angles), ranging from 0° (red) through 90° (yellow), 180° (green), 270° (blue) and back to 0° [21]. The colour coordinates were obtained from three different positions in each replicate (three replicates per sample) on both external surfaces and cross-sections of the burger samples.

The colour difference between redness (∆a*) and yellowness (∆b*), chroma (∆c*), and total colour differences (∆E*) in PB burgers at raw and cooked (75 °C) were evaluated considering Coles Classic beef burger as the reference sample using the following formula [22]

$$∆a^{*}=a_{2\;}^{*}-a_{1}^{*}$$

$$∆b^{*}=b_{2}^{*}-b_{1}^{*}$$

$$∆c^{*}=\sqrt{a_{2}^{*2}+b_{2}^{*2\;}}-\sqrt{a_{1}^{*2}+b_{1}^{*2}}$$

$$∆E_{ab}^{*}=\sqrt{{\left(L_{2}^{*}-L_{1}^{*}\right)}^{2}+{\left(a_{2}^{*}-a_{1}^{*}\right)}^{2}+{\left(b_{2}^{*}-b_{1}^{*}\right)}^{2}}$$

where a* redness; L* lightness; b* yellowness; $a_{1}^{*},b_{1}^{*}$ are the coordinates of the reference sample; $a_{1}^{*},\;b_{2}^{*}$ are the coordinates of the PB burger sample.

### 2.4. Shrinkage Analysis

The impact of internal temperature on shrinkage was evaluated based on the volume of the whole PB and AB burger patties at 75 °C internal temperature. The surface area of each patty was determined using grid paper (1 cm^2^ per square), while the height was measured at three distinct points using a vernier calliper (Starrett 125MEA-6/150 Vernier Caliper 6, Starrett Company, Beijing, China). The volume of the burgers was then calculated using the following formula [23].

$$Shrinkage\;\left({cm}^{3}\right)=surface\;area\;\left({cm}^{2}\right)\times height\;\left(cm\right)$$

### 2.5. Quantification Browning Intensity

The quantification of the browning intensity of all PB burgers at 75 °C internal temperature was analysed using Image J software 1.53t (National Institutes of Health, Bethesda, MD, USA). The browning intensity of PB burgers was compared with Coles Classic (reference) and chicken burgers, as they performed a red to white colour range in the raw state and a brown to less brown range in the cooked state, respectively. The standard photograph of each representative burger sample at 75 °C was formatted to 5020 pixels in grey scale for the analysis of each pixel with a scale of 0 to 255 (with 0 representing the colour black and 255 representing white pixels). The arithmetic mean of all pixel values reflects the intensity of the browning effect [24].

### 2.6. Data Analysis

Data was analysed using XLSTAT Software (Premium version (2023.1.6), Lumivero, Ltd., CO, USA) for the designing of colour maps, and shrinkage of the burgers. The variance analysis was done by ANOVA, and the means were compared with Tukey’s HSD at a significant level of *p* < 0.05. The visualization and interpretation of colour maps based on mean sample scores were performed using PCA (principal component analysis). The correlation of colour dimensions between PB and AB was also conducted using Pearson’s correlation.

## 3. Results and Discussion

### 3.1. External Colour Transition of Burger Samples Across Five Different Internal Temperatures

**Figure 1:**
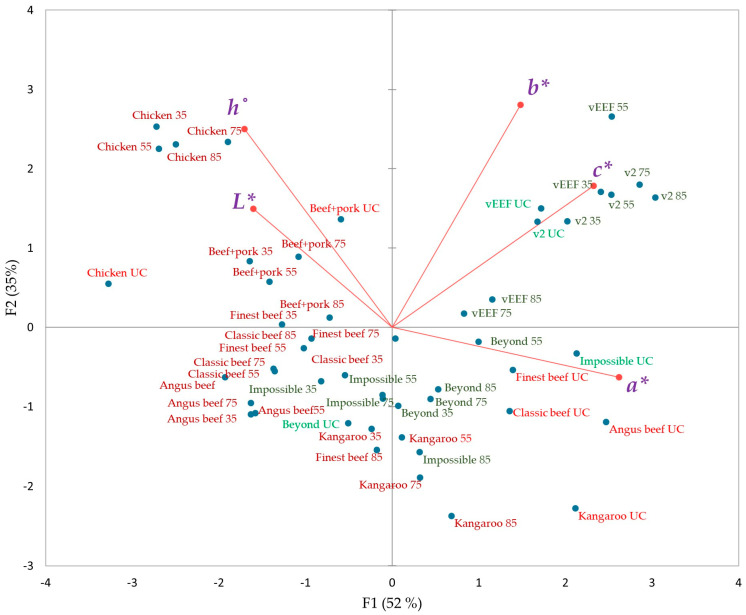
PCA biplot of external colour coordinates of ten animal-based and plant-based burgers in five different endpoint internal temperatures (UC: uncooked; 35: 35 °C; 55: 55 °C; 75: 75 °C; 85:85 °C) (PC1+PC2: 92%; *p* value < 0.0001) (light red signifies uncooked animal-based; dark-red signifies cooked animal-based; light-green signifies uncooked plant-based; dark-green signifies cooked plant-based) (L*: lightness; a*: redness; b*: yellowness; c*: chroma, *h*°: hue).

Generally, a food product’s visual appearance is determined by its light scattering and absorption properties [25]. The distribution of various particles and fibrous networks within meat contributes to light wave scattering, thereby leading to opacity. This opacity is primarily linked to the lightness (positive L*) value. Light absorption is associated with the presence of pigments within the product, predominantly interpreted as redness (positive a*) and yellowness (positive b*) [26]. The mean CIE L*, a*, b*, c*, and *h*° colour measurements for the external (cooked surface) of the 10 burger samples across five different internal temperatures are visualized by PCA in Figure 1 (PC1+PC2: 87%). The biplot effectively summarizes the multivariate colorimetric data, highlighting the major differences in colour dimensions between PB and AB burgers as a function of internal temperature. This dimensionality reduction facilitates the identification of clustering patterns and potential discriminative features associated with burger type and internal temperature. Principal component 1 (PC 1) (52%) is primarily influenced by a* (positive direction), while principal component 2 (PC 2), explaining 35% of the variance, is mainly driven by the *h*° and b* axis in the positive direction. The mean values of external and internal colorimetric values at each internal temperature level are also provided in Supplementary Tables S1 and S2.

**Figure 2:**
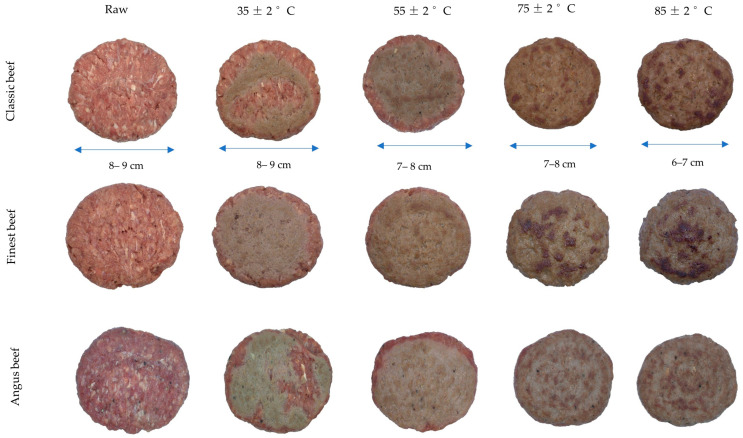

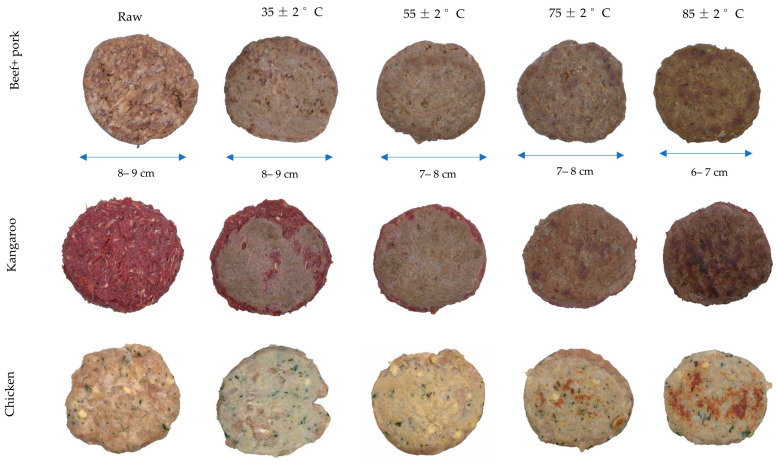
External colour transition of animal-based burgers at different internal temperatures (including the uncooked raw product for each sample).

All uncooked beef-based and kangaroo burgers clustered in lower-right quadrant of the biplot (Figure 1), due to the significantly higher positive a* (+16 to +19), low positive L* (+45 to +49), and *h*° (+36 to +42) values compared to chicken, beef+pork, v2food, vEEF, and Beyond burgers in the colorimetric coordinates (Table S1). This is undoubtedly due to the initial high redness of raw beef and kangaroo burgers, which can be observed in visual images, attributed to their high myoglobin and low-fat content [27] (Figure 2). The significantly higher positive L* (+53 to +59), *h*° (+63 to +70), and lower positive a* (+5 to +9) in chicken and beef+pork burgers, mainly explained by the lower myoglobin content, resulted position in the left-top quadrant of the biplot (Figure 1) [28]. Confirming this, a strong negative correlation was observed between L* and a* (*r* = −0.792) among uncooked animal-based burgers.

**Figure 3:**
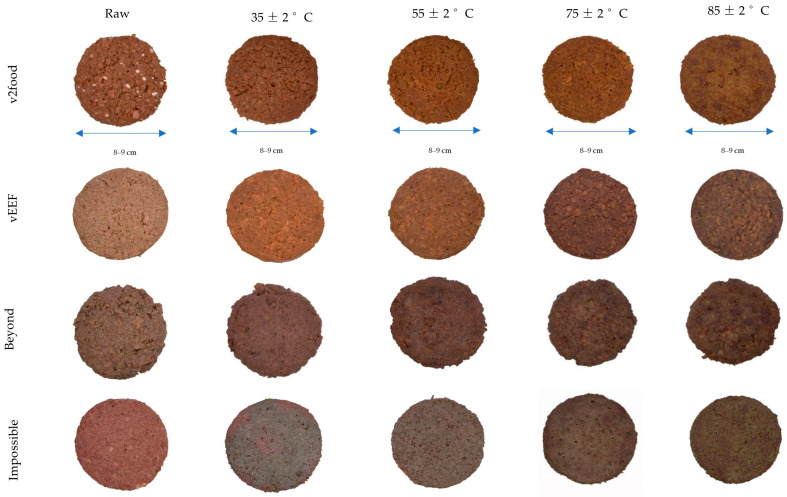
External colour transition of plant-based burgers at different internal temperatures (including the uncooked raw product for each sample).

Based on the colorimetric values, the uncooked PB burgers were distributed across the plot, ranging from the right (v2food, vEEF, and Impossible) to the lower-left (Beyond) quadrant (Figure 1). This distribution pattern in the uncooked state is attributed to the varying combinations of colour pigments, which influence light scattering and absorption within the matrix. Both v2food and vEEF are positioned along the b* and c* axis due to negative scores for ∆a* (−2.5 to −2.9), elevated positive range for ∆b* (+ 5.8 to +6.1), and high ∆E* values (+9.4 to +10.3), compared to Impossible PB burger (Table S3). As shown in processed visual images, the higher colour saturation of v2food and vEEF is attributed to the presence of chemically complex pigments from anthocyanins and their reflection of a broader range of wavelengths, leading to richer and more intense colours [29]. This is further explained by the strong negative correlation (*r* = −0.798) between L* and c* of PB burgers in their raw states. Among all PB burgers, Impossible had a significantly higher positive value of a* (+18), and the lowest colour differences (∆E*), closely resembling uncooked beef-based burgers. This rich “*juicy*, *bloody-red*” appearance and “*meaty*” flavour in Impossible burgers is enhanced by LegHb, which is structurally similar to mammalian myoglobin (Figure 3) [30]. Interestingly, the uncooked Beyond was placed on the lower-left side of the PCA biplot, predominantly allocated for the cooked beef and kangaroo burgers, due to its negative ∆a* compared to other PB burgers. This higher browning intensity of raw Beyond burgers can be explained by the presence of natural colour pigments (betanin and anthocyanin) different from other brands, and their high tendency to degrade into brown polymers, resulting in an elevated browning index (BI) [31,32].

As the internal cooking temperature increased, all beef-based burgers transitioned from the lower-right to the lower-left quadrant of the biplot (Figure 1). This transition is associated with the reduction in positive a* value, which is primarily attributed to the gradual degradation of myoglobin pigments and the Maillard reaction in AB burgers. At low internal temperatures (raw to 35 °C), myoglobin remains largely intact (oxymyoglobin or deoxymyoglobin), giving meat burgers a red to pink appearance (uneven browning). When the temperature reached “*well-done*” state at 75 °C, the presence of oxymyoglobin (Fe^2+^ bound to O_2_) and deoxymyoglobin (Fe^2+^ without O_2_) in the muscle is converted to metmyoglobin (Fe^3+^), which is brown [33]. Further heating leads to the formation of ferrihemochrome, a denatured heme-protein complex that contributes to the dull brown-grey colour in authentic meat burgers. Also, the Maillard reaction products between amino acids and reducing sugars at high internal temperatures facilitate the formation of melanoidins, which are brown pigments contributing to further brownish hue development of the burgers’ crust. [34,35,36]. Extensive protein denaturation and Maillard reaction occurrence, together with lipid oxidation and moisture loss, further intensify browning, which can be observed at 85 °C in meat burgers [34]. This gradual colour transition from red to brown and less lightness at increasing internal temperature in meat-based burgers could be observed in external visual images, and indeed it was strongly negatively correlated between a* and L* values (r = −0.967) at 75 °C internal temperature. Particularly, as shown in images, grey-brown colour development on the surface depends on the exposure to the surface of the grilling plate giving a mottled, inconsistent colouring especially evident at 35 °C for beef-based and kangaroo burgers (Figure 2). However, the lower myoglobin and different tissue structure in cooked chicken and beef+pork burgers remained in the same top-left cluster, with chicken burgers receiving significantly higher positive *h*° (+75 to +77) at higher internal temperatures [37].

At elevated internal temperatures, the v2food sample exhibited significantly higher positive chromaticity shifts, with ∆c* and ∆b* values reaching +10 and +8, respectively, resulting in a pronounced overall colour difference (∆E* ≈ +10) (Table S3). These values were notably greater than those observed in Beyond and Impossible burgers, which demonstrated ∆E* values in the range of +5 to +6, respectively. The enhanced colour change in v2food is primarily attributed to the thermal degradation of beetroot extract, leading to the formation of bright yellow betalamic acid and colourless cyclodopa 5-O-glucoside [12,38]. Among all PB burgers, only Impossible burgers followed the same colour transition trajectory as beef during cooking, imitating “*meaty-like*” visual appearance due to the production of browning polymers through Maillard reaction, water evaporation, and fat crystal melting over high internal temperatures [39,40]. The LegHb (derived from soy roots) in Impossible burgers, and myoglobin in animal meat, both contain a heme prosthetic group. In the raw state, Fe^2+^ binds with O_2_ to form oxymyoglobin, imparting a red hue. Upon heating, protein denaturation alters the heme environment, promoting oxidation of Fe^2+^ to Fe^3+^, thereby replicating the colour transition observed in beef. Additionally, LegHb catalyses Maillard reactions, converting amino acids and sugars into volatile aroma compounds and melanoidins, which enhance browning and flavour development [41].

### 3.2. The Internal Colour Transition of Burger Samples Across Five Different Internal Temperatures

**Figure 4:**
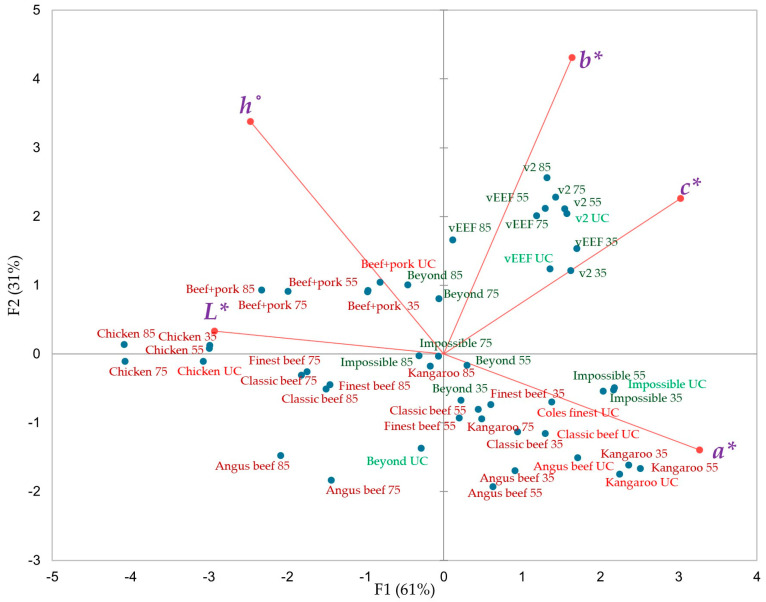
PCA biplot of internal colour coordinates of ten animal-based and plant-based burgers in five different endpoint internal temperatures (UC: uncooked; 35: 35 °C; 55: 55 °C; 75: 75 °C; 85; 85 °C) (PC1+PC2: 92%; *p* value < 0.0001) (light red signifies uncooked animal-based; dark-red signifies cooked animal-based; light- green signifies uncooked plant-based; dark green signifies cooked plant-based) (L*: lightness; a*: redness; b*: yellowness; c*: chroma, *h*°: hue).

**Figure 5:**
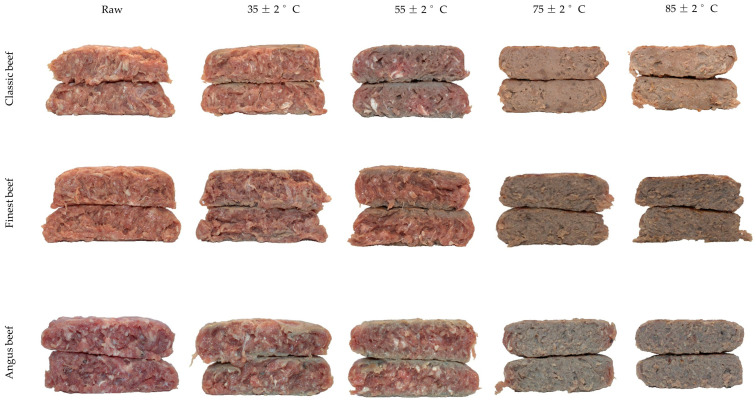

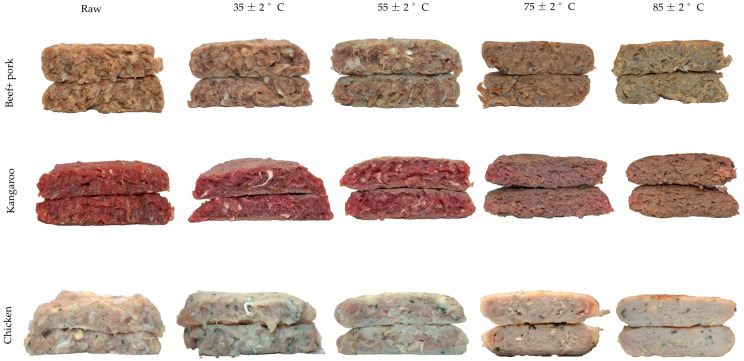
Internal colour transition of animal-based burgers at different internal temperatures (including the uncooked raw product for each sample).

The first two principal components of the PCA plot in Figure 4 account for 92% of the variation in the internal colorimetric values of ten burgers across five different internal temperatures. PC1, which accounts for 61% of the total variance, is primarily influenced by the a* value in the positive direction and L* in the negative direction. This suggests that the a* and L* are the dominant factors separating samples along PC1. PC2, explaining 31% of the variance, is mainly driven by b* and *h*° axis in the positive direction. The uncooked internal a* value was slightly higher positive (+17 to +19) in Coles Finest, Classic, and kangaroo burgers compared to their external positive a* (+16 to +17). This discrepancy may be attributed to induced surface oxidation, which leads to a lower positive a* value than internal oxidation, in vacuum-packaged food meat [42]. Among beef burgers, the Angus beef sample had significantly higher positive a* (+19) scores compared to classic (+17) and finest (+17) beef burgers at raw states (Table S2). Confirming this, a high-red intensified cross-section with connective tissues could be observed in Angus beef burgers than in other beef-based burgers (Figure 5). This could be attributed to the source of breed, muscle region, and other handling methods during meat processing [43]. The surface oxidation during thawing might also be attributed to the higher positive external L* (+53 to +59) value in chicken and beef + pork burgers than their internal values (+51 to +56).

At an internal temperature of 35 °C, the external colour transition of beef burgers shifted entirely from the right to the left of the plot, while the internal colour transition clustered in the lower-right of the biplot (Figure 4). This is likely due to the direct contact of the hot grill plate with the burger surface, causing rapid myoglobin denaturation on the surface rather than internally. In contrast, the internal colour shift of cooked beef and Impossible burgers from the right to the left-hand side of the biplot as internal temperatures vary follows a unique pattern, distinct from the external colour-changing pattern shown in Figure 1. This may be attributed to the transfer of heat waves through the burger matrix, enabling uniform thermal processing and progressive myoglobin denaturation. This gradual internal colour transition can be observed from a warm red centre (*rare*) at 35 °C and 55 °C to a more intense brown (*well-done*) state at 75 °C and 85 °C internal temperatures (Figure 5). Similarly, Sen et al. [44] reported that complete myoglobin denaturation occurs in muscles at 65 –80 °C. Chicken burgers exhibited a different pattern due to lower myoglobin concentration, and inclusion of flavour and texture-enhancing ingredients such as cheese particles and chopped herbs (Figure 5).

**Figure 6:**
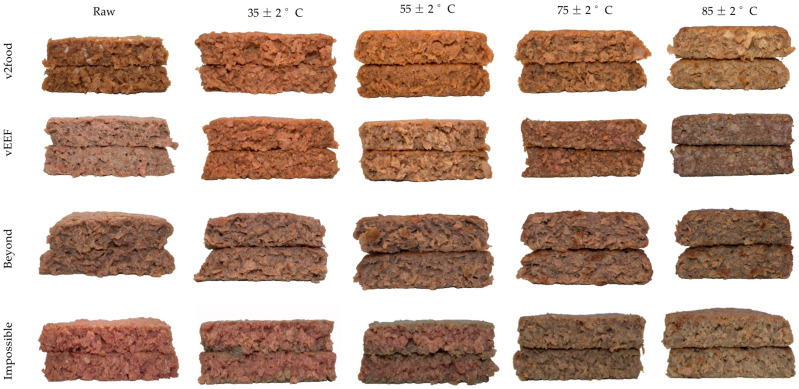
Internal colour transition of plant-based burgers at different internal temperatures (including the uncooked raw product for each sample).

Similar to the external, significantly higher ∆b*, ∆c*, and ∆E* values (Table S4) were observed in both v2food and vEEF in their raw and cooked states, corresponding to elevated b* and c* values. The internal yellowish-brown hue may result from caramelized sugar and malt extract, which enhance browning via caramelization and non-enzymatic browning (Maillard reaction) during cooking. Additionally, the presence of anthocyanin pigments as glycosylated compounds promotes the conversion to anthocyanidins during thermal processing. These anthocyanidins are highly susceptible to oxidative and ring-opening reactions, resulting in brown degradation products such as chalcones and phenolic acids [45,46]. Prolonged thermal degradation of anthocyanins facilitates their polymerization with other phenolic compounds or sugars, leading to the formation of brown polymeric pigments [47]. This strategic formulation results in a noticeable escalation in the intensity of the brown colour in PB burgers, both externally and in cross-sectional visual images, as the internal temperature of these meat analogues is increased (Figure 6). In contrast, Impossible burgers exhibited the minimum internal colour difference (∆a*, ∆b*, ∆c*, and ∆E*) at both raw and cooked states, attributed to the LegHb in the matrix.

**Figure 7:**
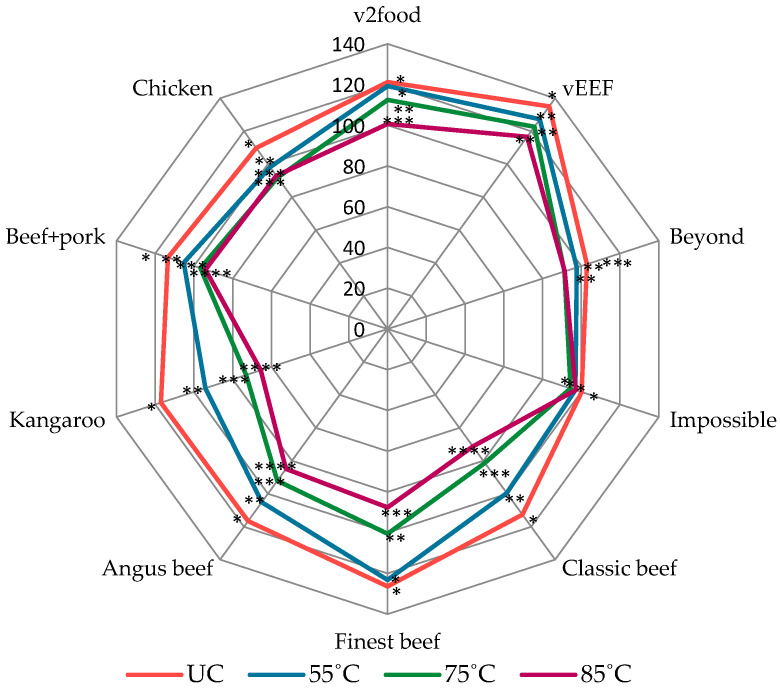
Impact of internal temperature on shrinkage (cm^3^) on animal-based and plant-based burgers. Significant differences among different internal temperatures of burger samples are indicated by * (*p* < 0.05). (UC: uncooked). (*, **, ***, **** shows the significant difference among each sample at different internal temperature).

Aside from colour changes, both uncooked AB and PB burgers exhibit moist, irregular cutting surfaces and a soft texture, attributable to the presence of oil and water within the matrix. As internal temperature rises during cooking, moisture is released, resulting in a transition to a firmer, drier texture with smoother cutting surfaces. The hypothesis was that the gradual increase in internal temperature leads to substantial cooking loss, promoting the release of exudates from the burgers. This, in turn, contributes to pronounced shrinkage and influences browning intensity through increased surface contact with the heated grill plate. Notably, AB burgers exhibited significantly higher fluid loss, likely due to the helix-to-coil transition of muscle proteins during heating, which causes tissue softening, followed by denaturation of actin and myosin, leading to structural shrinkage and fluid expulsion. In contrast, PB burgers maintained structural integrity under elevated internal temperatures, exhibiting relatively consistent shrinkage compared to AB burgers. This stability can be attributed to the presence of polymeric ingredients such as methylcellulose and modified starch, which possess strong water-binding capacity and contribute to the formation of a porous, cohesive network. (Figure 7) [23,48].

**Figure 8:**
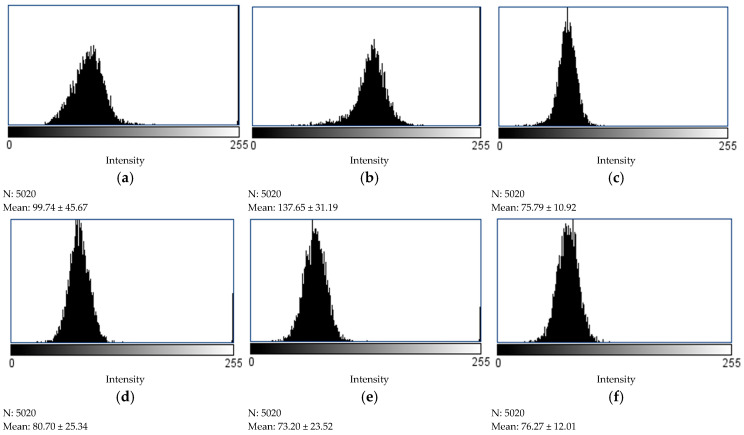
The degree of browning intensity quantified by determining the pixel density of processed images using Image J software (version 1.53t) (**a**) Coles Classic (beef); (**b**) Chicken; (**c**) v2food; (**d**) vEEF; (**e**) Beyond; (**f**) Impossible burger.

The browning intensity of Coles Classic (beef), Chicken, and PB burgers is shown in Figure 8 based on the associated pixel intensities of standard processed images. The beef sample exhibits a wider browning intensity (99.74 ± 45.66) compared to PB burgers, which is associated with the shrinkage of myofibrillar proteins during cooking. This structural contraction (Figure 7) leads to irregular surface morphology, resulting in non-uniform heat exposure and consequently variable browning compared to PB burgers. In contrast, chicken burgers displayed higher pixel intensity values (137.69 ± 37.19), indicative of a lighter surface colour, primarily due to their lower myoglobin content. The intact morphology of PB burgers at high internal temperature resulted in visually similar browning intensity with less wide distribution compared to the beef sample. However, among PB burgers, the Beyond burgers demonstrated the lowest pixel intensity (73.20 ± 23.52), corresponding to a more pronounced brown hue, as visually evident at the 75 °C internal temperature. Interestingly, the v2food sample (75.79 ± 10.92) showed slightly higher intensity than the vEEF burger (80.70 ± 25.33), likely due to the combined effect of caramelized sugar and betanin. These findings suggest that browning intensity is influenced not only by colour additives but also by the structural characteristics of the burger matrix, including the role of binding agents in heat transfer and surface uniformity. Particularly, the gelation behaviour of PB burgers due to methylcellulose maintains a uniform surface structure even at elevated temperatures, promoting consistent browning compared to AB burgers.

Overall, the colour transition of PB burgers varied by brand, primarily due to the presence of different pigment combinations, concentrations, the composition of the base materials, and chemical affinity in the matrix. Arguably, the visual distinction between AB and PB is observed in their uncooked state. This difference is primarily attributed to the presence of myoglobin pigments in authentic meat, whereas the inherent instability of natural colorants in PB formulations often results in a comparatively brownish hue. Szpicer et al. [49] reported that spray-dried encapsulated anthocyanin incorporation can provide colour within an acceptable range, while also enhancing the antioxidant capacity in gluten and soy-free plant-based meat analogues. The application of colour inhibitors, microencapsulation, and innovative pigmentation processes might ensure stability and enhance the desired colour tone in PB meat analogues at raw and cooked states [50].

## 4. Conclusions

This study examines the visual appearance and colour transitions of commercial PB and AB burgers across varying internal temperatures. Results indicate that premature browning in red meat burgers occurs at relatively low internal temperatures, primarily due to direct and uneven contact with the hot grilling surface. At 75 °C, beef and kangaroo burgers reached a “*well-done*” state, characterized by decreased positive a* values and increased positive *h*° values, attributable to myoglobin denaturation, the Maillard reaction, and lipid oxidation. Among PB burgers, the Impossible burger exhibited the highest positive a* and the lowest ∆E* in the raw state, with a browning transition most closely resembling that of beef. AB burgers showed significant reductions in size and shape during cooking, driven by myofibrillar protein denaturation and shrinkage, which also contributed to browning intensity. In contrast, PB burgers largely retained their original shape, likely due to the structural stability provided by methylcellulose binding. The v2food and vEEF samples displayed the highest positive c*, b*, and ∆E* values in both raw and cooked states, resulting in a more orange-brown appearance. These findings suggest that the current application technique of anthocyanins and betanin does not successfully imitate authentic beef colour transitions.

Future research should integrate sensory panel evaluations of colour transitions across internal temperatures with image-based analysis to provide a comprehensive understanding of visual changes during cooking. Reducing positive b*, c*, and ∆E* values, enhancing the stability of pigments and structural integrity, are critical when employing natural colour-based predictive models for future meat analogues. Achieving targeted meat-like colour transitions requires a systematic understanding of pigment–matrix interactions, which will be essential for optimizing visual sensory attributes and ensuring consumer acceptance of future plant-based meat products.

## Figures and Tables

**Table 1:** Sample details, including brand and ingredients of commercial PB and AB burgers (as indicated on the product label).

Commercial Name (Sample)	Binder	Colouring Agent	Protein and Fat (g per 100 g)	RCM
Plant-based burgers				
vEEF plant-based premium beef burger	MC, PS, cocoa butter	beetroot powder, malt extract	12.8; 13.5	each side 3 min 30 s (74 °C IT)
v2food plant-based burger	MC, modified cornstarch, carrageenan	beetroot powder, caramelized sugar	17.7; 14.6	each side 3–4 min
Beyond burger (plant-based patties)	MC, PS	beetroot juice, apple extract, pomegranate concentrate	17; 19	each side 4 min. (75 °C IT)
Impossible burger patties (made from plants)	MC, modified starch	soy leghemoglobin (GM)	16.7; 11.4	each side 2 min (71 °C IT)
Animal-based burgers				
Coles finest beef	PS	paprika and spices as flavour enhancers	15.7; 10.3	each side 5 min.
Coles classic beef	MS, PS	spices as flavour enhancers	12.7; 20.8	10–12 min
Angus beef	MS, PS	flavour enhancers	16; 15	14 min
Beef and pork	PS	flavour enhancers & colourants	13; 16.5	each side 4–5 min
Chicken	PS	flavour enhancers	16.2; 9.1	10–12 min
Kangaroo	rice flour	flavour enhancers	18; 1.9	each side 4–5 min

MC: methylcellulose, PS: potato starch, MS: maize starch, RCM: recommended cooking method, IT: internal temperature.

## Data Availability

The original contributions presented in this study are included in the article/Supplementary Material. Further inquiries can be directed to the corresponding author.

## Supplementary Materials

The following supporting information can be downloaded at: https://www.mdpi.com/article/10.3390/foods14213616/s1, Table S1: External colorimetric mean values of plant and animal meat-based burgers; Table S2: Internal colorimetric mean values of plant and animal meat-based burgers; Table S3: External colour differences in plant-based burgers at raw and 75 °C internal temperature; Table S4: Internal colour differences in plant-based burgers at raw and 75 °C internal temperature
