# Supplementary material for: Colour Transition Dynamics of Commercial Plant- and Animal-Based Meat Analogues
Source: Foods. 2025 Oct 23;14(21):3616. doi: 10.3390/foods14213616 (PMC12609363; doi:10.3390/foods14213616)
Supplement: Supplementary file 1 [file foods-14-03616-s001.zip › foods-3898186-supplementary.pdf]

Title: Colour Transition Dynamics of Commercial Plant and Animal-based Meat Analogues

Supplementary tables

Table S1. External colorimetric mean values of plant and animal meat-based burgers

Category	Lightness (*L**)	Redness (*a**)	Yellowness (*b**)	Chroma (*c**)	Hue (h°)
Veef 55	42.8^{klmno}	14.70^{defg}	25.82^{a}	29.74^{a}	59.9^{ghi}
V2 75	34.10^{wxyz}	15.40^{def}	24.27^{ab}	28.75^{ab}	57.61^{ijklm}
Veef 35	42.57^{lmno}	15.81^{cde}	22.93^{bcd}	27.858^{abc}	55.43^{lmnopq}
Beef+pork UC	53.01^{c}	9.40^{opqrst}	18.15^{ghi}	20.56^{hijklm}	62.76^{g}
Veef UC	42.99^{jklmn}	13.57^{ghi}	21.03^{def}	26.94^{bcd}	56.92^{jklmn}
V2 85	30.62$^{aa\ ab\ ac}$	15.48^{cdef}	24.26^{ab}	28.78^{ab}	57.29^{ijklm}
V2 UC	41.84^{mnop}	13.95^{fgh}	21.29^{cde}	25.42^{cde}	56.7^{jklmn}
V2 35	38.32^{rstu}	14.31^{efg}	21.79^{cde}	26.23^{cde}	56.9^{jklmn}
Chicken 75	52.78^{c}	4.49^{x}	20.25^{efg}	19.02^{ijklmno}	75.38^{d}
Chicken 85	53.21^{c}	3.45^{xy}	18.31^{ghi}	18.60^{lmnopqr}	77.30^{cd}
Chicken 55	58.50^{b}	3.66^{xy}	17.36^{hijkl}	17.76^{nopqrs}	77.40^{bcd}
Coles finest UC	49.59^{d}	16.44^{bcd}	15.19^{lmnopqr}	22.39^{fgh}	42.59^{wx}
Impossible UC	45.01^{hijk}	17.77^{ab}	16.64^{ijklmno}	24.34^{efg}	43.12^{wx}
Veef 85	32.31$^{yz\ aa}$	11.91^{ijk}	18.73^{ghi}	22.19^{gh}	57.52^{ijklm}
Veef 75	32.82$^{xyz\ aa}$	11.36^{jklm}	17.92^{hij}	21.03^{hijkl}	57.86^{hijklm}
Angus UC	45.16^{hijk}	19.41^{a}	13.94^{pqrstuvw}	24.77^{def}	36.18^{y}
Beef+pork 75	43.45^{ijklm}	7.15^{uvw}	16.81^{hijklmn}	18.27^{mnopqr}	67.30^{ef}
Coles classic UC	45.49^{hi}	15.93^{cde}	14.09^{pqrstuvw}	21.42^{hi}	40.80^{x}
Beyond 55	34.92^{vwx}	12.50^{hij}	16.96^{hijklm}	21.07^{hijk}	53.63^{opqr}
Coles finest 75	36.36^{uvw}	9.98^{lmnop}	15.94^{jklmnop}	18.90^{jklmnop}	57.90^{hijklm}
Beef+pork 35	48.44^{def}	6.66^{w}	15.45^{lmnopq}	16.82^{opqrstuv}	66.69^{f}
Chicken 35	62.33^{a}	2.56^{y}	14.77^{mnopqrs}	14.99^{vwxyz}	80.22^{ab}
Beef+pork 55	45.19^{hij}	6.88^{vw}	15.53^{lmnopq}	16.99^{opqrstuv}	66.10^{f}
Coles finest 35	49.46^{d}	8.42^{pqrstuv}	14.18^{pqrstuvw}	16.50^{pqrstuv}	59.32^{hij}
Chicken 35	61.84^{a}	2.14^{y}	14.57^{opqrstu}	14.98^{vwxyz}	81.13^{a}
Beef+pork 85	38.92^{qrst}	8.17^{qrstuvw}	15.69^{klmnopq}	17.46^{nopqrstu}	62.43^{g}
Beef+pork 55	43.47^{ijklm}	6.56^{w}	15.52^{lmnopq}	17.04^{opqrstuv}	67.10^{ef}
Coles classic 85	40.99^{nopq}	8.01^{rstuvw}	14.40^{pqrstuv}	16.98^{opqrstuv}	60.65^{gh}
Coles finest 55	44.88^{hijkl}	8.58^{pqrstu}	14.01^{pqrstuvw}	16.43^{qrstuv}	58.17^{hijkl}
Beyond 85	28.85$^{ac\ ad}$	10.89^{jklmno}	15.14^{mnopqr}	18.78^{klmnopq}	55.27^{mnopq}
Beyond 75	30.78$^{aa\ ab\ ac}$	11.20^{jklmn}	14.64^{nopqrst}	18.32^{mnopqr}	54.06^{nopq}
Chicken UC	58.86^{b}	4.53^{x}	12.22^{vwxyz}	13.04^{z}	69.85^{e}
Kangaroo UC	34.38^{wxy}	17.18^{bc}	12.15^{wxyz}	21.28^{hij}	34.22^{y}
Beyond 35	39.75^{pqrs}	11.28^{jklm}	13.64^{qrstuvwxy}	17.78^{nopqrs}	50.00st
Coles classic 55	45.66^{ghi}	8.14^{rstuvw}	12.71^{stuvwxyz}	15.24^{tuvwxyz}	57.79^{hijklm}
Coles classic 75	45.10^{hijk}	7.98^{stuvw}	12.93^{stuvwxyz}	15.12^{uvwxyz}	58.24^{hijkl}
Impossible 35	40.44^{opqr}	9.49^{opqrs}	13.91^{pqrstuvw}	16.84^{opqrstuv}	55.68^{klmnop}
Impossible 75	31.8$^{z\ aa\ ab}$	9.70^{mnopqr}	14.07^{pqrstuvw}	17.07^{nopqrstuv}	56.08^{klmno}
Impossible 55	37.76^{stu}	10.45^{klmno}	13.90^{pqrstuvwx}	17.39^{nopqrstuv}	53.05^{pqr}

Angus 85	49.14^{de}	7.24^{uvw}	11.70^{xyz}	13.82^{xyz}	58.48^{hijk}
Kangaroo 55	38.16^{rstu}	11.41^{jkl}	12.41^{uvwxyz}	$17.63^{nopqrst}$	48.11^{tu}
Coles classic 35	44.80^{hijkl}	9.84^{lmnopq}	$12.86^{stuvwxyz}$	16.31^{rstuvw}	55.24^{mnopq}
Beyond UC	46.10^{fgh}	10.87^{jklmno}	12.12^{wxyz}	$16.28^{rstuvwx}$	48.10^{tu}
Kangaroo 35	42.39^{mno}	11.23^{jklmn}	12.32^{vwxyz}	$16.68^{opqrstuv}$	48.48^{tu}
Angus 35	46.93^{efgh}	7.93^{stuvw}	11.37^{z}	13.94^{wxyz}	55.69^{klmnop}
Impossible 85	$28.10^{ad\ ae}$	10.70^{klmno}	$13.10^{rstuvwxyz}$	$16.91^{opqrstuv}$	50.77^{rst}
Angus 55	46.43^{fgh}	8.00^{stuvw}	11.01^{z}	13.720^{yz}	55.04^{mnopq}
Angus 75	44.8^{hijkl}	7.79^{tuvw}	11.15^{z}	13.92^{wxyz}	55.60^{klmnop}
Kangaroo 75	$29.75^{ab\ ac\ ad}$	10.86^{jklmno}	$12.48^{tuvwxyz}$	$16.43^{pqrstuv}$	46.720^{uv}
Kangaroo 85	25.99^{ae}	11.82^{jk}	11.61^{yz}	$16.58^{opqrstuv}$	43.94^{vw}
Coles finest 85	$29.56^{ab\ ac\ ad}$	9.58^{nopqrs}	$12.60^{stuvwxyz}$	$15.73^{stuvwxy}$	52.70^{qrs}
Pr > F(Model)	<0.0001	<0.0001	<0.0001	<0.0001	<0.0001
Significant	Yes	Yes	Yes	Yes	Yes

UC: uncooked; 35: 35°C, 55: 55°C, 75:75°C, 85:85°C

Table S2. Internal colorimetric mean values of plant and animal meat-based burgers

Category	Lightness (L^*)	Redness (a^*)	Yellowness (b^*)	Chroma (c^*)	Hue ($h^°$)
V2 UC	43.11^{lmnopq}	13.79^{qrs}	24.12$^{ac\ ad}$	27.785^{w}	60.23^{kl}
Veef 55	43.52^{opqr}	12.96^{opq}	24.20$^{ac\ ad}$	26.96^{vw}	61.86^{m}
Veef 85	49.03^{xy}	10.78^{k}	21.60^{aa}	24.14^{tu}	63.50^{no}
V2 75	41.55^{hijklmn}	12.50^{ad}	24.52^{ad}	27.54^{vw}	62.94^{mn}
V2 55	42.03^{ijklmnop}	13.35^{pqr}	24.16$^{ac\ ad}$	27.69^{lm}	61.36l^{m}
V2 85	41.27^{ghijklm}	11.72^{klmn}	25.47^{ae}	27.27^{vw}	65.00^{p}
Veef 35	42.72^{jklmnopq}	14.89^{tu}	22.78^{ab}	27.22^{i}	56.82^{i}
Beef+pork UC	51.28$^{z\ aa}$	9.30^{j}	18.75^{xy}	20.86^{nop}	64.72^{op}
Veef 75	41.71^{hijklmno}	12.12^{mno}	23.46$^{ab\ ac}$	26.41^{v}	62.70^{mn}
Veef UC	42.91^{klmnopq}	13.12^{opq}	20.62^{z}	27.34^{vw}	56.48^{i}
Coles finest UC	46.45^{tuv}	17.29^{wx}	16.13^{pqrs}	23.65^{t}	42.99^{d}
Beef+pork 35	46.84^{uvw}	7.82^{gh}	17.85^{vwx}	19.50^{ijklm}	67.06^{q}
Beef+pork 75	52.83^{aa}	5.07^{cd}	16.18^{qrst}	17.04^{g}	73.52^{x}
Beef+pork 85	50.84^{yz}	5.45^{cde}	16.60^{rstu}	17.50^{gh}	71.49^{vw}
Impossible 55	41.18^{ghijkl}	18.17^{xy}	16.95^{stuv}	24.85^{u}	43.00^{d}
Impossible UC	40.50^{fghi}	18.48^{yz}	17.18^{tuv}	25.24^{u}	42.96^{d}
V2 35	35.20^{bc}	13.11^{opq}	21.34$^{z\ aa}$	25.05^{u}	58.44^{j}
Impossible 35	38.84^{ef}	18.03^{xy}	17.01^{stuv}	24.81^{u}	43.43^{d}
Coles finest 35	45.95^{tu}	13.93^{qrst}	14.94^{klmno}	20.43^{mno}	47.03^{ef}
Coles finest 55	46.21^{tu}	14.34^{rst}	14.56^{hijklm}	20.46^{mno}	45.99^{e}
Angus UC	43.84^{pqrs}	18.69^{yz}	13.92^{fghijk}	23.01st	37.34^{b}
Beef+pork 55	43.33^{nopq}	7.00^{fg}	17.49^{uvw}	18.84^{ijk}	68.72^{rs}
Coles classic UC	43.37^{nopq}	16.56^{vw}	14.69^{ijklm}	22.06^{qrs}	41.30^{c}
Coles classic 35	45.54^{stu}	15.74^{uv}	14.35^{ghijkl}	21.70^{pqr}	42.42^{cd}
Beyond 75	40.62^{fghi}	9.44^{j}	19.16^{y}	19.65^{ijklm}	62.30^{mn}
Chicken 35	60.34$^{ad\ ae}$	6.36^{ef}	14.41^{hijkl}	15.28^{e}	68.97st
Beyond 85	39.55^{g}	7.50^{gh}	18.41^{wxy}	19.58^{ijklm}	67.40^{qr}
Kangaroo 75	42.35^{ijklmnop}	13.52^{pqrs}	15.13^{lmnop}	20.29^{lmno}	48.23^{f}
Chicken 35	59.40$^{ac\ ad}$	5.62^{de}	13.59^{efghi}	14.93^{de}	69.39st
Kangaroo 85	43.15^{mnopq}	10.72^{k}	15.76^{nopqr}	19.08^{ijkl}	56.44^{i}
Coles classic 55	44.52^{qrst}	12.69^{nop}	14.41^{hijkl}	19.12^{ijkl}	47.57^{f}
Chicken 55	56.73$^{ab\ ac}$	4.82^{cd}	12.93^{cdefg}	14.73^{de}	70.97^{uvw}
Coles finest 75	49.50^{xyz}	7.22^{fg}	13.89^{fghijk}	15.66^{ef}	62.52^{mn}
Beyond 55	39.99^{fgh}	11.38^{klm}	16.22^{qrst}	19.83^{jklmn}	54.91^{h}
Chicken 85	63.16$^{ae\ af}$	3.8^{ab}	12.23^{bcde}	12.56^{ab}	75.17^{y}
Angus 35	45.37^{rstu}	15.92^{v}	13.32^{defg}	20.06^{klmn}	37.41^{b}
Coles classic 75	49.53^{xyz}	7.15^{fg}	13.63^{efghi}	15.39^{e}	62.85^{mn}
Kangaroo 55	35.20^{bc}	19.26^{z}	13.77^{efghi}	23.68^{t}	35.60^{a}
Chicken 55	57.09$^{ab\ ac}$	4.52^{bc}	12.80^{cdef}	13.80^{cd}	70.41^{tuv}
Chicken 85	62.59$^{ae\ af}$	3.12^{a}	12.120^{bcde}	12.52^{ab}	76.05^{y}
Coles finest 85	48.29^{vwx}	8.02^{ghi}	13.75^{efghij}	15.91^{ef}	59.74^{jk}
Chicken UC	56.105^{ab}	4.62^{bcd}	12.47^{cde}	13.92^{cd}	69.95^{stu}
Kangaroo 35	33.39^{ab}	18.60^{yz}	13.87^{efghi}	22.54^{rs}	37.77^{b}
Chicken 75	63.84^{af}	3.15^{a}	12.10^{bcde}	12.03^{ab}	71.78^{vw}
Beyond 35	40.84^{ghi}	11.80^{lmn}	14.81^{jklmn}	18.91^{ijk}	51.37^{g}

Chicken 75	63.48[af]	3.22[a]	11.11[abc]	11.57[a]	72.35[wx]
Coles classic 85	48.60[wx]	7.88[ghi]	13.6[efghi]	15.72[ef]	59.22[jk]
Impossible 75	37.22[de]	9.42[j]	15.91[opqr]	18.49[hi]	59.39[jk]
Impossible 85	35.73[cd]	8.44[hij]	15.47[mnopq]	17.46[gh]	62.07[mn]
Kangaroo UC	31.93[a]	18.25[xyz]	13.53[efgh]	21.29[opq]	38.25[b]
Angus 55	43.14[mnopq]	14.48[st]	12.02[abcd]	18.74[ij]	38.48[b]
Angus 85	49.93[xyz]	7.14[fg]	10.48[ab]	12.57[ab]	54.96[h]
Angus 75	46.66[uvw]	8.88[ij]	10.13[a]	13.12[bc]	50.14[g]
Beyond UC	41.07[ghijk]	10.92[kl]	12.09[bcde]	16.59[fg]	49.96[g]
Pr > F(sample)	<0.0001	<0.0001	<0.0001	<0.0001	<0.0001
Significant	Yes	Yes	Yes	Yes	Yes

UC: uncooked; 35: 35˚C, 55: 55˚C, 75:75˚C, 85:85˚C

Table S3. External colour differences in plant-based burgers at raw and 75˚C internal temperature

Sample	*Δa**	*Δb**	*Δc**	*ΔE**
v2food (raw)	-2.48^{d}	6.10^{b}	3.03^{c}	10.32^{a}
vEEF (raw)	-2.87^{d}	5.84^{b}	4.55^{b}	9.39^{a}
Beyond (raw)	-5.57^{e}	-3.07^{e}	-6.11^{e}	7.39^{b}
Impossible (raw)	1.33^{b}	1.45^{c}	1.95^{c}	5.07^{c}
v2food (75˚C)	5.42^{a}	8.34^{a}	9.85^{a}	10.27^{a}
vEEF (75˚C)	2.38^{b}	2.99^{c}	4.13^{c}	7.44^{b}
Beyond (75˚C)	1.22^{b}	-1.30^{d}	-0.57^{d}	5.96^{bc}
Impossible (75˚C)	-0.28^{c}	-1.87^{d}	-1.82^{d}	5.04^{c}

*Δa**: redness difference; *Δb**: yellowness difference; *Δc**: chroma difference; *ΔE**: Total colour difference (Coles finest beef burgers used as the reference sample at both raw and 75˚C internal temperature). Different superscripts within a column indicate significant differences ($p < 0.05$).

Table S4. Internal colour differences in plant-based burgers at raw and 75˚C internal temperature

Sample	*Δa**	*Δb**	*Δc**	*ΔE**
v2food (raw)	-3.50^{d}	7.99^{b}	9.45^{c}	4.14^{b}
vEEF (raw)	-4.17^{d}	4.50^{c}	7.30^{d}	3.69^{b}
Beyond (raw)	-6.37^{e}	-4.04^{e}	9.32^{c}	-7.06^{d}
Impossible (raw)	0.36^{c}	0.88^{d}	6.78^{d}	1.60^{c}
v2food (75˚C)	5.28^{a}	10.64^{a}	14.33^{a}	11.89^{a}
vEEF (75˚C)	4.90^{a}	9.57^{a}	13.36^{ab}	10.76^{a}
Beyond (75˚C)	2.22^{b}	5.27^{c}	10.59^{c}	4.00^{b}
Impossible (75˚C)	2.19^{b}	2.03^{d}	12.68^{b}	2.83^{bc}

Δa*: redness difference; Δb*: yellowness difference; Δc*: chroma difference; ΔE*: Total colour difference (Coles finest beef burgers used as the reference sample at both raw and 75˚C internal temperature). Different superscripts within a column indicate significant differences ($p < 0.05$).
